# The Value of Private Patient Information in the Physician-Patient Relationship: A Game-Theoretic Account

**DOI:** 10.1155/2012/847396

**Published:** 2012-12-31

**Authors:** Kris De Jaegher

**Affiliations:** Utrecht University School of Economics, Utrecht University, 3584 EC Utrecht, The Netherlands

## Abstract

This paper presents a game-theoretical model of the physician-patient relationship. There is a conflict of interests between physician and patient, in that the physician prefers the patient to always obtain a particular treatment, even if the patient would not consider this treatment in his interest. The patient obtains imperfect cues of whether or not he needs the treatment. The effect of an increase in the quality of the patient's private information is studied, in the form of an improvement in the quality of his cues. It is shown that when the patient's information improves in this sense, he may either become better off or worse off. The precise circumstances under which either result is obtained are derived.

## 1. Introduction

All across developed countries, in the aftermath of the financial crisis of 2008, cash-strapped governments are currently seeking ways to cut their budgets. Given the rising costs of health care, this seems a good area for cutting the budget, as suspicion may arise that physicians prescribe unnecessary treatments. A good way to cut the budget would then seem to provide patients with more information so that they can better assess their health status, and in such a way that they avoid unnecessary treatment. In fact, one would then hope that the drastically increased opportunities for patients to obtain information through the Internet (see [1]), would increasingly pose a constraint on physician's ability to overprescribe without any necessity to intervene. The purpose of this paper is to show, using a simple game-theoretic model, that such increased patient information does not necessarily decrease health care expenditure and may actually make patients worse off.

The question on whether or not the hypothesis that physicians overprescribe treatment (known as the supplier-induced demand hypothesis [2]) is confirmed has attracted a huge literature in health economics). While the discussion continues on whether supplier-induced demand exists and whether it can be observed continues, the literature clearly shows that physicians respond to incentives [3]. The natural conclusion is then that conflicts of interest will arise between physician and patient, and that the physician will not always prescribe treatments which a hypothetical patient with the same information as the physician would consider in his own interest. The question we seek to address is whether better patient information can counter the conflict of interest between physician and patient. We analyze this question using a simple game-theoretic model of the physician-patient relationship, which is an extension of de Jaegher and Jegers [4].

While the vast majority of the supplier-induced demand literature in health economics is empirical, part of this literature has attempted to give a theoretical underpinning to the hypotheses formulated in the literature. Microeconomic models, and in particular game theory, provide tools that are apt to construct such theories. Physician and patient are assumed to be rational players who maximize their expected payoffs, given the expected behaviour of the other player. Examples include [4–7]. This health economics literature is closely related to a wider literature in economics that analyzes the relation between expert and client in general (for an overview, see [8]). Specific to the expert-client relation is that the expert not only sells services to the client, but also advises the client on which services the client needs, potentially creating a conflict of interest between expert and client. A problem to the client is that he may not be able to experience ex post whether or not the expert's advice was in his interest. For instance, if a patient is cured after treatment, this may not be due to the treatment, but because the patient's disease is self-curing. The difference between the theoretical expert-client literature and the theoretical health economics literature is that the former has put more emphasis on market mechanisms, where the experts can freely set their prices, whereas the latter has put more emphasis on fixed prices, which are more realistic in a physician-patient setting.

The paper in the literature closest to the current paper is Xie et al. [7], who provide an extension to de Jaegher and Jegers [4] with the purpose of studying the impact of improved patient information. In Xie et al., the patient either needs a treatment *A* (e.g., a cheap treatment) or a treatment *B* (e.g., an expensive treatment). The physician prefers that the patient always obtains the expensive treatment. The authors assume two types of patients, namely one type who is more likely to need the cheap treatment, which they call a well-informed patient, and another type who is less likely to need the cheap treatment, which they call an ill-informed patient. Depending on the frequency of each type of patient, either a mixed equilibrium exists where what the authors consider as the well-informed patient always refuses the expensive treatment and the so-called ill-informed patient is indifferent between accepting and refusing the expensive treatment, or a mixed equilibrium where the well-informed patient is indifferent between accepting and refusing the expensive treatment, whereas the ill-informed patient accepts it. An increase in the probability that one or both types of patients need the cheap treatment (which if the physician's least preferred treatment) is considered by the authors as an improvement in their information. In this interpretation of patient information, in the current mixed equilibrium, let what the authors consider as the well-informed patient be indifferent between accepting or refusing the expensive treatment, whereas the ill-informed patient refuses the expensive treatment. Let it now be the case that the patient who is less likely to need the cheap treatment, whom the authors consider as the ill-informed patient, become even more likely to need the cheap treatment, which the authors consider as an improvement in his information. If this so-called improvement in information is large enough, then this causes the mixed equilibrium to switch to one where the authors' well-informed patient accepts expensive treatment, whereas the authors' ill-informed patient is indifferent about accepting or refusing it. As in the new mixed equilibrium the physician is more likely to unnecessarily prescribe the expensive treatment, both patient types are now worse off. At the same time, smaller changes in the patient's information that do not lead to a switch form one mixed strategy equilibrium to another, do not change the probability that the physician overprescribes.  Appendix B contains a summary of Xie et al.'s analysis, for easy comparison.

A disadvantage of Xie et al.'s model is that the patient's probability of needing the cheap treatment is at the same time considered as his degree of information, so that it is not possible to disentangle whether one is looking at the effect of a change in the probabilities of the states of the world (i.e., a change in the incidence of disease), or at a change in the patient's information. In this respect, unnoticed by Xie et al., a fragmented and dispersed literature in game theory and economics has studied the effect of the value of public information and of private information. In the games studied in this literature, players' payoffs depend on the one hand on the actions of themselves and other players, and on the other hand on the state of the world. Players have a common prior over the probabilities of the states of the world (e.g., the incidence of disease). A player's private information is information that he possesses about the state of the world, where the content of this information is not observed by other players (though other players are assumed to know that the patient possesses private information). Public information is information about the state of the world that is common knowledge to all players. The literature studies improvements in the quality of this information, where probability of the states of the world do not change when information changes. The literature shows that both improved public and private information may have either positive or negative information (see [9–13]). While in decision theory [14], more information can never make a decision maker worse off, in the interactive decision theory studied in economics and game theory, it can. ^1^ It should be stressed here that there is no single model or theory of the value of public or private information. Rather, a negative value of information has been observed for very diverse specific examples of games, where such a negative value of information may each time occur for very different reasons.

In the light of the literature on the possible negative value of private information studied in the literature, we add to de Jaegher and Jegers' [4] model of the physician-patient relationship, private information for the patient. Following the literature on the value of information, we assume our patient and physician to have a common prior over the probability that the states of the world occur. Additionally, the patient observes an imperfect cue of his true state, where this cue is not observed by the physician. The quality of the patient's information can now be changed in our model without changing the probability of the states, so that contrary to Xie et al. [7] we can study the pure effect of an improvement in information. This leads us to results about the negative value of patient information that differ from Xie et al. In our model, either the patient receives an imperfect cue that he needs the expensive treatment (an expensive cue), or an imperfect cue that he needs the cheap treatment (a cheap cue)—where it does not make sense in our model to call a patient with one cue better or worse informed than a patient with another cue. If the physician has weak incentives for overprescribing, then a mixed equilibrium exists where the patient with an expensive cue buys expensive treatment after getting it recommended, whereas the patient with a cheap cue is indifferent about buying expensive treatment or no treatment after getting an expensive recommendation. In this case, we show that making the patient's cues more accurate makes the patient better off. If the physician has strong incentives for overprescribing, then a mixed equilibrium exists where the patient with an expensive cue is indifferent about buying an expensive treatment or buying no treatment after an expensive recommendation, whereas the patient with a cheap cue does not buy treatment. In this case, for a range of smaller improvements in the quality of the patient's cues, the patient is made worse off the better quality of his cues. This is because the only manner in which the physician can in equilibrium keep the patient with a better expensive cue indifferent between buying the expensive treatment or not buying treatment after an expensive recommendation, is by overprescribing more often. Contrary to what is the case in Xie et al., in our model such an effect does not require a switch from one type of mixed equilibrium to another type of mixed equilibrium. On the contrary, in our model when the increase in the quality of the patient's information is large enough to induce a switch to the other type of mixed equilibrium, the patient is better off with more information. That a sufficiently large increase in the quality of the patient's private information has positive value, can simply be seen that in the hypothetical case where the quality of the patient's cues becomes perfect, so that he has the same information as the physician, the patient simply obtains the right treatment in the right state of the world. 

The paper is structured as follows.  Section 2 contains our simple model of the physician-patient relationship.  Section 3 derives the mixed equilibria of this model, and studies the effect on these equilibria of improvements in the patient's private information. We end with some conclusions in Section 4. 

## 2. Material and Methods

Following de Jaegher and Jegers [4], the following simple Bayesian extensive form game between a physician (she) and a patient (he) is considered. At stage 1, Nature decides with probability *π*_*C*_ that state of the world *C* occurs (the patient is best off with a cheap treatment), and with probability *π*_*E*_ that state of the world *E* occurs (the patient is best off with an expensive treatment), where *π*_*C*_ + *π*_*E*_ = 1. The state of the world is observed by the physician, but not by the patient. At stage 2 (which is absent in de Jaegher and Jegers (2001)), Nature lets the patient observe an imperfect cue of the state of the world. In particular, when the true state of the world is *j* = *C*, *E*, the patient observes cue *i* with probability *π*(*i* | *j*), where *i* = *C*, *E*, and where *i* may or may not be different from *j*. The physician does not observe the patient's cues. ^2^ We assume that *π*(*i* | *i*) ≥ *π*(*i* | *j*), so that in each state, the patient is not more likely to receive a wrong than a right cue. Applying Bayes' rule, for the patient observing cue *i*, the updated probability of being in state of the world *i* equals *π*_*i*_ *π*(*i* | *i*)/[*π*_*i*_ *π*(*i* | *i*) + *π*_*j*_ *π*(*i* | *j*)]. It follows that cue *i* is informative to the patient if this updated probability is larger than *π*_*i*_, which is the case if *π*(*i* | *i*) > *π*(*i* | *j*). Thus, given our assumption that *π*(*i* | *i*) ≥ *π*(*i* | *j*)), we both allow for the case where the patient gets informative cues (*π*(*i* | *i*) > *π*(*i* | *j*)), and does not get informative cues (*π*(*i* | *i*) = *π*(*i* | *j*)).

At stage 3, the physician, having observed the state of the world decides on which treatment to prescribe to the patient. It should be stressed here that the physician does not observe which cue was obtained by the patient she faces, making this cue private information to the patient. ^3^ In each state of the world, the physician may either prescribe a *C* treatment (cheap treatment), or an *E* treatment (expensive treatment). The physician may thus in each state be seen as either honestly informing the patient about the state of the world, or misinforming the patient.

At stage 4, after having observed his imperfect cue of the state of the world, but not the state of the world itself, and after having observed the physician's recommendation, the patient either decides to buy the expensive treatment from the physician (action *E*), the cheap treatment from the physician (action *C*), or no treatment from the physician (action 0). In each state of the world *i*, the patient may thus get either treatment *C*, treatment *E*, or no treatment, which is denoted as 0. ^4^ We note here that the possibility that the patient does not buy treatment from the physician complicates the game, but is necessary to ensure that at least some information transmission takes place from physician to patient. To see why, suppose that buying the cheap or buying the expensive treatment are the only actions available to the patient. Then as soon as prescribing the expensive treatment leads the patient more often to buy the expensive than the cheap treatment, the physician, who is assumed to prefer that the patient gets the expensive treatment, will always prescribe it. Only when a third action is available in the form of not buying treatment, can the physician be disciplined.

Finally, both players obtain their payoffs. These payoffs reflect all aspects of each player's preferences, including their attitudes towards risk. The payoff of the physician is denoted as Π(*i* | *j*), where *j* is the state of nature (*C* or *E*), and where *i* is the action taken by the patient (*C*, *E* or 0). We normalize the physician's payoffs such that Π(0 | *E*) = Π(0 | *C*) = 0 (it can be checked that assuming different values for Π(0 | *E*) = Π(0 | *C*) does not make any difference for the results, but only makes the calculations more complicated), and that Π(*E* | *j*) > Π(*C* | *j*). The latter implies that the physician prefers that the patient obtains the expensive treatment, whatever the state of the world. Further, we assume that the physician's payoffs are such that Π(*C* | *C*)/Π(*E* | *C*) > Π(*C* | *E*)/Π(*E* | *E*). Thus, while the physician is always better off if the patient obtains the expensive treatment, her payoff of the patient getting the cheap treatment relative to her payoff of the patient getting the expensive treatment, is larger in state *C* than in state *E*. Such an assumption is plausible, as the interests of the physician and patient need not be 100% opposed, in the sense that the physician perceives less benefits from prescribing an *E* treatment when knowing that the patient would not consider this in his own interests^5^. The assumption ensures that if the patient's treatment decisions are such that the physician is indifferent about whether or not to prescribe expensive treatment in state *C*, she strictly prefers to prescribe expensive treatment in state *E*.

The payoff of the patient is denoted as *U*(*i* | *j*), where *j* again denotes the state of the world (*C* or *E*), and *i* denotes the action taken by the patient (get a cheap treatment (*C*), get an expensive treatment (*E*), or get no treatment (0)). We assume that *u*(*E* | *E*) > *u*(0 | *E*) > *u*(*C* | *E*); *u*(*C* | *C*) > *u*(0 | *C*) > *u*(*E* | *C*). This means that the patient prefers to get the *C*(*E*) treatment in state *C*(*E*), and prefers to get no treatment (0) to getting the wrong treatment. Moreover, the patient's payoffs are assumed to be such that *π*_*i*_ *π*(*k* | *i*)[*u*(*i* | *i*) − *u*(0 | *i*)]/*π*_*j*_ *π*(*k* | *j*)[*u*(0 | *j*) − *u*(*i* | *j*)] < 1 for *i*, *j* = *C*, *E*, *i* ≠ *j*, and *k* = *i*, *j*, meaning that a patient without physician information strictly prefers not to buy any treatment, independent of his cue. If without further physician information, the patient prefers to buy the *E* treatment, it is easy to see that the physician does not have any incentive to make her prescriptions informative. Also, if without further physician information, the patient prefers to buy the *C* treatment, it will never occur that the patient does not buy treatment, which is the decision that the patient needs to be taking to discipline the physician. It should be noted that the decision not to buy any treatment means a decision not to buy any treatment from the physician, but leaves open the possibility that the patient consults another physician (even though the strategic interaction arising with second opinions is not modeled here).

All aspects of the game are common knowledge to the players (e.g., the patient knows the probabilities of the states; while the physician does not know what are the patients' cues, the physician knows that the patient observes cues with specific probabilities^6^; etc). The physician's strategy consists of a prescription strategy, namely a plan on how often to prescribe the two types of treatment in each state of the world. The patient's strategy consists of a treatment decision strategy, namely a plan on how often to get the cheap treatment, the expensive treatment, or no treatment at all, for any given prescription by the physician. A best response strategy is a strategy that maximizes a player's expected payoff, given the strategy employed by the other player. Physician and patient strategies that are mutual best responses describe a perfect Bayesian equilibrium. The underlying reasoning is that each player, given his or her beliefs about the other player's strategy, does what is best for him or her to do, given the other player's strategy, where additionally these beliefs are fulfilled in equilibrium. In order to assess how well either patient or physician does with an equilibrium, we calculate the patient's or physician's expected payoff, that is, what payoff he or she can expect on average ex ante, before finding out the state of the world. 

## 3. Results and Discussion

### 3.1. Patient without Information (*π*(*i* | *i*) = *π*(*i* | *j*) for *i* = *C*, *E*, *i* ≠ *j*)

For expositional reasons, we here shortly repeat in this section the case already treated in de Jaegher and Jegers [4], where the patient does not receive informative cues, so that *π*(*i* | *i*) = *π*(*i* | *j*). Effectively, stage 2 of the game as described in Section 2, where the patient observes cues of the true state of the world, is omitted now.

Proposition 1 shows the existence of a mixed equilibrium for this game, where we note that all proofs in this paper can be found in Appendix A. For the proof that this is the *only* equilibrium where the physician transmits information to the patient, we refer to de Jaegher and Jegers [4]. Intuitively, it cannot be that the physician's recommendation to buy an expensive treatment is only done when expensive treatment is necessary, as otherwise the patient would always follow the recommendation, in turn inducing the physician to always prescribe the expensive treatment. Further, it cannot be the case either that the physician always prescribes the expensive treatment when it is not necessary, since otherwise the patient would never follow the advice. It follows that in any informative equilibrium, when the cheap treatment is necessary, the physician must sometimes recommend the expensive treatment, and sometimes the cheap treatment. Further, it cannot be the case that the patient always follows the physician's advice to buy an expensive treatment, as otherwise the physician would always prescribe this. But at the same time, it should not be the case in an informative equilibrium that the patient never follows up such advice. It follows that the patient must mix between following up the physician's advice to buy an expensive treatment, and not following it up. ^7^

**Proposition 1**

Consider the physician-patient game presented in Section 2, and assume that *π*(*i* | *i*) = *π*(*i* | *j*) for *i*, *j* = *C*, *E* and *i* ≠ *j*, meaning that the patient's cues are completely uninformative. Then the game has a mixed equilibrium where:

 (i) the patient always follows a recommendation to buy the cheap treatment and randomizes between accepting and refusing a recommendation to buy the expensive treatment;
 (ii) the physician always prescribes the expensive treatment when it is necessary, and randomizes between prescribing the cheap and the expensive treatment when only the cheap treatment is necessary.

**Figure 1:**
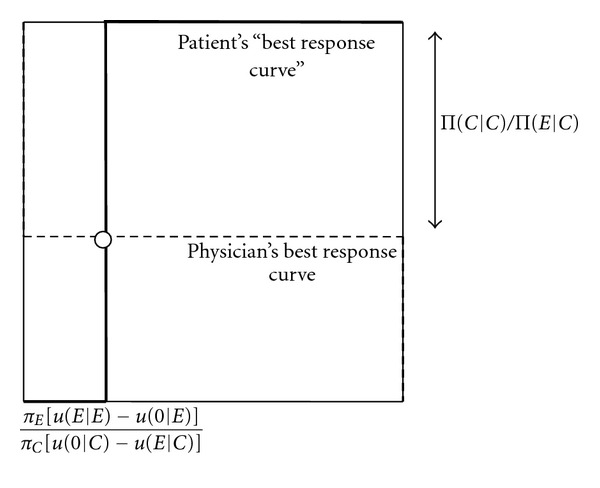
Best response curves and mixed equilibrium without patient information. *p* (probability that patient does not buy treatment when getting an expensive treatment recommended). *q* (probability that the physician recommends an unnecessary expensive treatment).

Figure 1 explains the intuition of Proposition 1. The dashed curve represents the physician's best response curve, in terms of how often she should recommend the expensive treatment when only a cheap treatment is necessary (*q*, on the *X* axis), as a function of the probability that the patient who needs only a cheap treatment does not buy treatment when getting a recommendation to buy an expensive treatment (*p*, on the *Y* axis). For a high probability of not buying treatment (*p* high), the physician prefers to recommend the cheap treatment (*q* = 0), as the high probability makes it better for her to recommend the cheap treatment, where this recommendation is always followed by the patient. For an intermediate probability of not buying treatment (*p* = 1 − Π(*C* | *C*)Π(*E*∣*C*)^−1^), the physician is indifferent between recommending the cheap and the expensive treatment. This is reflected by the dashed horizontal line, showing that the physician may recommend the expensive treatment with any probability. Finally, for a low probability that the patient does not buy treatment (*p*low), the physician always recommends the expensive treatment (*q* = 1).

The solid curve is the patient's best response curve. If the physician overprescribes infrequently (*q* low), the patient prefers to follow any recommendation to buy the expensive treatment (*p* = 0). For a particular probability that the physician overprescribes, the patient is indifferent between following the physician's recommendation or not buying treatment, as indicated by the vertical solid line. Finally, if the physician overprescribes with high probability (*q* high), the patient prefers not to buy any treatment (*p* = 1). The equilibrium is obtained at the intersection of the best response curves.

### 3.2. Patient with Information (*π*(*i* | *i*) > *π*(*i* | *j*) for *i* = *C*, *E*, *i* ≠ *j*)

We now come to the main contribution of this paper, namely extending the model of de Jaegher and Jegers [4] to the case where the patient has imperfect private information (in the sense that this information is not observed by the physician) about his true state of the world. In order to allow easy comparison with the results of Xie et al. [7], Appendix B contains a summary and explanation of their results. In Proposition 2, we first show the existence of two types of mixed equilibria in our extended game. ^8^

**Proposition 2**

Consider the physician-patient game presented in Section 2, and assume that *π*(*i* | *i*) > *π*(*i* | *j*) for *i*, *j* = *C*, *E* and *i* ≠ *j*, meaning that the patient's cues are informative. Thena mixed equilibrium exists where the physician recommends the expensive treatment when it is necessary, and randomizes between recommending the expensive and cheap treatment when only the cheap treatment is necessary. Further, in this mixed equilibrium:

 (i) if Π(*C* | *C*)/Π(*E* | *C*) > *π*(*E* | *C*) (i.e., the physician recommends treatment *C* in state *C* if the patient who receives an *E* recommendation does 0 after a *C* cue and *E* after an *E* cue), a patient who receives a recommendation to buy an expensive treatment follows the recommendation after an expensive cue, and randomizes between buying an expensive treatment and buying no treatment after a cheap cue;
 (ii) if Π(*C* | *C*)/Π(*E* | *C*) < *π*(*E* | *C*) (i.e., the physician recommends treatment *E* in state *C* if the patient who receives an *E* recommendation does 0 after a *C* cue and *E* after an *E* cue), a patient who receives a recommendation to buy an expensive treatment does not buy treatment after a cheap cue, and randomizes between buying expensive treatment and buying no treatment after an expensive cue.

**Figure 2:**
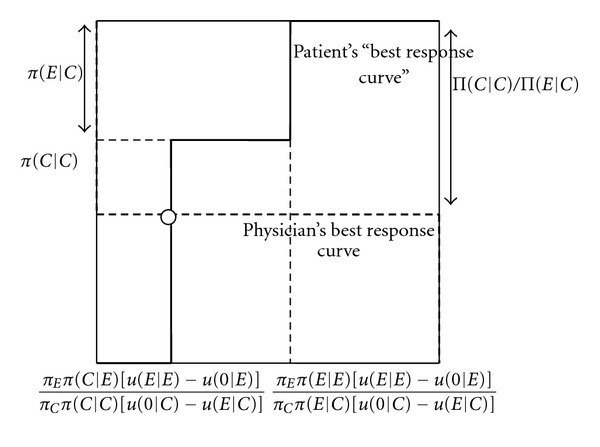
Best response curves and mixed equilibrium with patient information: patient who accepts (refuses) expensive treatment upon expensive (cheap) cue disciplines physician. *p*_*C*_ (probability that patient does not buy treatment when getting an unnecessary expensive treatment recommended). *q* (probability that the physician recommends an unnecessary expensive treatment).

**Figure 3:**
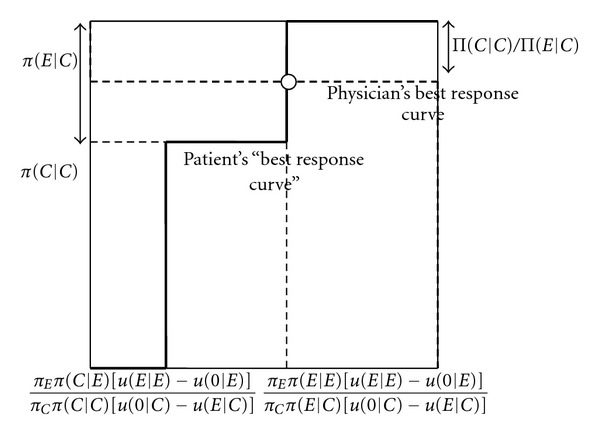
Best response curves and mixed equilibrium with patient information: patient who accepts (refuses) expensive treatment upon expensive (cheap) cue does not discipline physician. *p*_*C*_ (probability that patient does not buy treatment when getting an unnecessary expensive treatment recommended). *q* (probability that the physician recommends an unnecessary expensive treatment).

Figures 2 and 3 explain the intuition of Proposition 2. The best response curve of the physician has exactly the same form as in Figure 1. From the perspective of the physician, it only matters how often the patient on average does not buy treatment when getting an unnecessary expensive treatment recommended. The physician only cares about the overall probability that the patient accepts or refuses.

The solid curve represents the patient's best response curve, in terms of how often he does not buy treatment when an unnecessary expensive treatment is recommended. If the physician only overprescribes infrequently (*q* low), the patient always follows the recommendation to buy an expensive treatment, whatever his cue (*p*_*C*_ zero). As the physician overprescribes more often (*q* increases), one hits a frequency of recommendation where the patient who observes a cue that he needs the cheap treatment, is indifferent about whether to buy the expensive treatment or no treatment, whereas the patient who observes a cue that he needs the expensive treatment strictly prefers to buy the expensive treamtent. In this case, in the state where cheap treatment is efficient, overall the patient may not buy any treatment when expensive treatment is recommended, with any probability between zero and the probability that he receives a cheap cue (0 ≤ *p*_*C*_ ≤ *π*(*C* | *C*)). This is reflected by the vertical solid line most to the left. As the frequency with which the physician overprescribes is further increased, the patient with a cue that he needs cheap treatment does not buy treatment when getting an expensive treatment recommended, whereas the patient with a cue that he needs an expensive treatment follows the recommendation. In this case, the overall probability that the patient does not follow up a recommendation to buy the expensive treatment if he needs the cheap treatment is simply equal to the probability that he receives a cue that he needs a cheap treatment in this case (*p*_*C*_ = *π*(*C* | *C*)). As the frequency with which the physician overprescribes is increased even further (*q* increases further), a frequency is reached where the patient with a cue that he needs an expensive treatment is indifferent between buying treatment or not buying treatment when getting an expensive recommendation. In this case, in the state where cheap treatment is efficient, overall the patient may not buy treatment when an unnecessary expensive treatment is recommended with any probability between the probability that she receives a cheap cue and 1 ((*π*(*C* | *C*) ≤ *p*_*C*_ ≤ 1)). This is reflected by the vertical solid line most to the right. Finally, the frequency with which the physician overprescribes reaches such a level that the patient does not buy treatment when getting recommended an unnecessary expensive treatment (*p*_*C*_ = 1).

As shown in Figures 2 and 3, the physician's best response curve may intersect the patient's best response curve in either of the two vertical parts of the patient's best response curve, so that depending on the parameters, two different mixed equilibria are obtained. Which type of mixed equilibrium is obtained depends on the level of the physician's payoff when the patient gets the expensive treatment in the state where the cheap treatment is efficient, relative to the level of her payoff if the patient gets the cheap treatment. If the physician's payoff when the patient gets the expensive treatment is very high, the patient will have to not buy treatment very often to discipline the physician, meaning that the patient getting an expensive recommendation never buys a treatment upon a cheap cue, and randomizes upon an expensive cue. If the physician's payoff when the patient gets the expensive treatment is less high, it suffices that the patient follows the expensive recommendation upon an expensive cue, and randomizes upon a cheap cue. 

We now use the result in Proposition 2 to study the effect of improved patient information on the expected payoff of the patient and the physician. This leads us to Proposition 3.

**Proposition 3**

Consider the physician-patient game presented in Section 2, and assume that *π*(*i* | *i*) > *π*(*i* | *j*) for *i*, *j* = *C*, *E* and *i* ≠ *j*, meaning that the patient's cues are informative. Consider first the hypothetical case where the patient's private information is improved such that patient has the same information as the physician. In this case both patient and physician are better off than with a mixed equilibrium. Consider next smaller increases in the patient's private information. Then:

 (i) if Π(*C* | *C*)/Π(*E* | *C*) > *π*(*E* | *C*) (i.e., the physician recommends treatment *C* in state *C* if the patient who receives an *E* recommendation does 0 after a *C* cue and *E* after an *E* cue), such increases in patient information make both physician and the patient better off,
 (ii) if Π(*C* | *C*)/Π(*E* | *C*) < *π*(*E* | *C*) (i.e., the physician recommends treatment *E* in state *C* if the patient who receives an *E* recommendation does 0 after a *C* cue and *E* after an *E* cue) small increases in information such that the mixed equilibrium continues to be of type (ii) in Proposition 2, make the physician better off but the patient worse off; large increases in information such that the mixed equilibrium changes into one of type (i) in Proposition 2, make both physician and patient better off.

To explain the results in Proposition 3, we first point out that the patient is obviously better off when perfectly knowing the states of the world. This also applies to the physician, because in any mixed equilibrium, the physician in the state where the cheap treatment is efficient, obtains the same payoff as if she would never overprescribe. It follows that the physician's payoff when the patient has full information is only changed in the state where the expensive treatment is efficient. As with complete information the patient always buys the expensive treatment when this is efficient, the physician in better off when the patient is fully informed. Intuitively, if the patient has little information, given that the physician has incentives to overprescribe, she will not consider the physician's recommendation to buy the expensive treatment very convincing, and she may then not buy any treatment. If the patient is fully informed, however, the patient will at least always buy the expensive treatment when it is necessary. The reader may ask now: given that the physician is better off when the patient is fully informed, why does the physician not inform the patient in the first place? The problem is that the physician may not be able to make the information available to the patient showing that he needs the expensive treatment, simply because the patient cannot process such information. Further, informing the patient by simply telling him what treatment he needs is not credible: if the patient would believe the physician to always recommend the treatment in his best interest, the physician would always recommend the expensive treatment.

**Figure 4:**
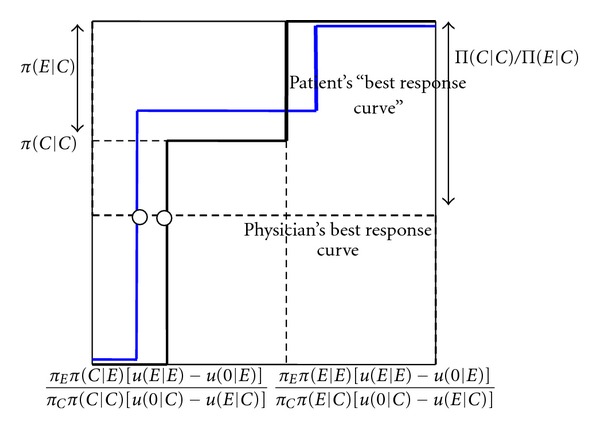
Small increase in patient information increases patient and physician payoff. *p*_*C*_ (probability that patient does not buy treatment when getting an unnecessary expensive treatment recommended). *q* (probability that the physician recommends an unnecessary expensive treatment).

We further look at small improvements in the patient's information. The intuition for Proposition 3(i) can be explained by means of Figure 4. An increase in patient information decreases the critical probability of the physician overprescribing, for which the patient with a cheap cue is indifferent between buying expensive treatment and not buying it when getting a recommendation to buy expensive treatment. Further, it increases the probability that a patient who does not follow a recommendation to buy expensive treatment when receiving a cheap cue, and follows it when receiving an expensive cue, does not buy treatment overall. This is reflected by the new, blue best response curve of the patient. As it is the patient with a cheap cue who is indifferent between whether or not to follow the recommendation, it follows that after the improvement in patient information it continues to be for such a cue that the patient is indifferent. As illustrated in Figure 4, in equilibrium the physician therefore overprescribes less often. As the physician's best response curve does not change when the patient's information improves, the overall probability that the patient does not follow a recommendation to buy unnecessary expensive treatment remains the same. Since the patient is indifferent between following and not following a recommendation to buy the expensive treatment, his payoff is at exactly the same level as in the case where he always follows the recommendation. It follows that the patient's expected payoff only depends on the probability that the physician overprescribes. As this decreases, the patient becomes better off. Further, as in the mixed equilibrium, the physician is equally well off when always prescribing the cheap treatment when only such a treatment is necessary, the physician's payoff in this state does not change with patient information. In the state where the expensive treatment is necessary, overall the patient follows the recommendation more often because the expensive cue occurs more often. The physician's payoff therefore increases for better patient information.

**Figure 5:**
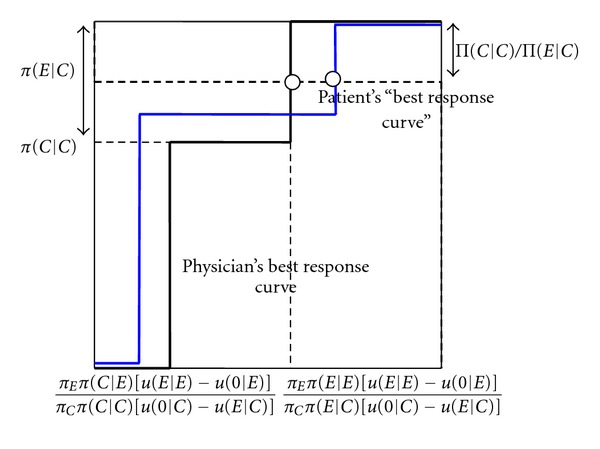
Small increase in patient information increases physician payoff but decreases patient payoff. *p*_*C*_ (probability that patient does not buy treatment when getting an unnecessary expensive treatment recommended). *q* (probability that the physician recommends an unnecessary expensive treatment).

**Figure 6:**
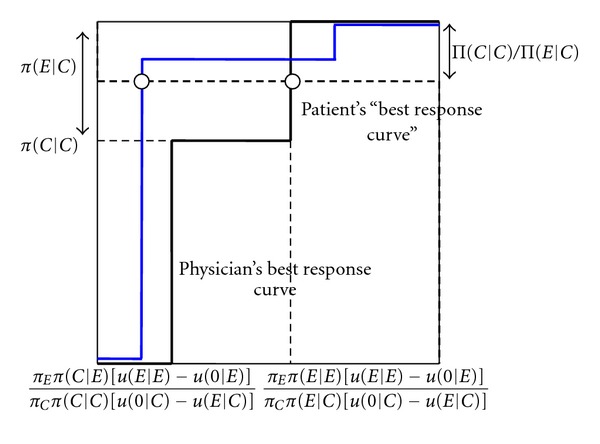
Large increase in patient information increases physician and patient information (switch from one type of mixed equilibrium to the other). *p*_*C*_ (probability that patient does not buy treatment when getting an unnecessary expensive treatment recommended). *q* (probability that the physician recommends an unnecessary expensive treatment).

The intuition for Proposition 3(ii) is obtained from Figures 5 and 6. An increase in patient information increases the critical probability of the physician overprescribing, for which the patient with an expensive cue is indifferent between buying expensive treatment or not buying treatment when getting a recommendation to buy the expensive treatment. Further, it again increases the probability that a patient who does not follow the recommendation when receiving a cheap cue and follows it when receiving an expensive cue, does not buy treatment overall. In Figure 5, the increase in information does not lead to a change in the type of mixed equilibrium played. As shown there, this means an increase in the probability that the physician overprescribes. Intuitively, as the patient's expensive cue is more reliable, the physician needs to overprescribe more often to make the patient with such a cue indifferent between following and not following the recommendation. It is this effect that leads to the surprising result that more information makes the patient worse off. The physician's payoff, however, increases as the patient observes the expensive cue more often. In Figure 6, the increase in information leads to a change in the type of mixed equilibrium, so that the patient always follows the recommendation to buy an expensive treatment when getting an expensive cue, and randomizes between following and not following when getting a cheap cue (note that this does not lead to a change in the overall probability of the patient not following the recommendation in the cheap state, because the cheap cue in this case occurs more often, and the expensive cue less often). Because of the reduction in the probability that the physician overprescribes, the patient becomes better off. The physician is also better off because the patient more often follows the recommendation to buy an expensive treatment when it is necessary. The perverse effect that better patient information makes the patient worse off thus only applies to smaller changes in information, such that the type of mixed equilibrium does not change.

The reader may further ask: if improved information makes the patient worse off, why does the patient not simply shut his ears to such information? Consider the situation without improved information, but let extra information be freely available to the patient. Let patient and physician play the original mixed equilibrium, before the improvement in information (see Proposition 1). Then, given the physician's current strategy, the best response of the patient is to use the extra information. But this will in turn lead the physician to change her strategy, leading to the new outcome. It should be stressed that it is not the case that the patient does not anticipate that the physician will change her strategy. It is just that if they play the original equilibrium before the patient seeks better information, the patient cannot credibly commit himself to ignoring such information.

We now sketch the following extension of the game, given the possibility that improved private information makes the patient better off. Assume that the physician is able to improve the patient's private information, by making information available to the patient. It should be stressed that this is not evidence about the patient's specific state of the world, but merely information that allows the patient to make an imperfect self-diagnosis, where the result of this self-diagnosis continues to be private information to the patient. ^9^ Concretely, the game set out in Section 2 is extended with a stage 0, where the physician can make information available to the patient, which allows the patient to obtain cues about the true state of the world, once Nature has chosen on this. After this, the game proceeds as before, where it continues to be the case that the physician does not observe the patient's cues. Then clearly, by the above, it is systematically in the interest of the physician to make such information available, if the costs attached to making such information available are not too large. However, the information made available may make the patient worse off. In this case, we obtain a model of information pushing, as suggested by Hirshleifer [15]. While the patient is not demanding information as it makes him worse off, information is pushed by the physician. This is simply done by making the information available, in which case the patient cannot credibly commit to not using the information, by the argument given above.

## 4. Discussion and Conclusion

In this paper, we have treated a highly stylized model of the physician-patient relationship that allows us to assess the impact of improved private information of the patient. Important to note here is that such an analysis only makes sense in an institutional environment where alternatives are available to a patient who refuses to follow the physician's recommendations—such as getting a second opinion from another physician, self-treatment, or simply abstaining from treatment. For instance, if without extra information, the patient prefers to buy the treatment most preferred by the physician, there is no incentive for the patient to refuse treatment, and the physician's prescription is not informative. Having additional information does not change anything for the patient.

Before interpreting the results, it should further be stressed that a mixed equilibrium is quite a special object: in any such equilibrium, in spite of the fact that each player is indifferent about what to do, he or she randomizes in a very specific manner, in order to make the other player indifferent about what to do. Yet, as pointed out by Harsanyi [16], any mixed equilibrium can be considered as replicating behaviour in a more complicated model, where mixing occurs because there is a continuum of types of each player. Thus, the individual patient may face a random physician from a population of physicians, who differ according to their tendency to overprescribe, such that for a given probability that the patient refuses, some physician types overprescribe and some do not. In a similar way, any physician may face a random patient from a population of patients, who differ according to their payoff when refusing treatment, such that some patients accept treatment while others refuse. The current model can now be considered as a limit point of a more general model, where the variance in the preferences of patients and physicians approaches zero.

Finally, the model we have employed is the simplest possible setting in which the effect of having informed patients in an environment where physicians have an incentive to overprescribe, can be analysed. The patient may in reality face many possible states of the world and many treatment options. It is conceivable to construct a more complex model allowing for such features, but this is outside of the scope of this paper. In any case, any tractable model will continue to be highly stylized, and will not necessarily lead to additional insights to those already treated in this paper. The aim of game-theoretic modeling can never be to make a completely realistic model of reality. Rather, in game theory, in tractable models, insights are gained into effects that would not have been understood without such game-theoretic modeling, and which identify circumstances under which such effects may be at work in the far more complex environment of a real-world setting.

In particular, for cases where the patient simply does not buy treatment from the physician unless the physician's prescription is sufficiently informative, we identify precise conditions under which having more information makes the patient worse off. First, it needs to be the case that the patient who is just willing to follow the physician's advice to buy expensive treatment, given the probability with which the physician overprescribes, is the patient who gets cues indicating that he needs expensive treatment, so that the patients who get cues that they only need cheap treatment do not follow the physician's recommendation to buy expensive treatment. This in turn is only possible if the physician is highly motivated to prescribe unnecessary expensive treatment. Second, the increase in the quality of the patient's information needs to be relatively small. A patient who gets a cue that he needs expensive treatment then becomes even more inclined to follow the physician's advice to buy expensive treatment. Such a patient will only remain indifferent about whether to follow the physician's advice if the physician is even more likely to overprescribe. A larger increase in the quality of the patient's information, however, leads to a switch to another type of equilibrium, where it is the patient who gets a cue that he needs cheap treatment who is just still willing to follow the physician's advice to buy expensive treatment. As this patient is less willing to flow the physician's recommendation, on average, the patient becomes better off with such a radical increase in the quality of his information.

The analysis leads to the following policy implications. Consider a government that suspects that physicians are overprescribing, and attempts to limit overprescribing by making information available to patients that makes them more able to assess their own condition. Then the government needs to realize that providing such information to patients may in particular circumstances make matters worse. The government should only make such information available, first, if the critical patient, who is currently just still willing to follow the physician's prescription to buy expensive treatment, is a patient who has reasons to believe that he is more likely to need cheap rather than expensive treatment. This occurs if the incentives of physicians to overprescribe are currently weak. Second, if the physician's incentives to overprescribe are on the contrary strong, so that the critical patient is one who in fact considers it relatively likely that he does need expensive treatment, then the government can only improve the average patient's situation with a substantial improvement in the patient's information, so that the patient with a cue that he does not need expensive treatment becomes the critical patient. Further, a government who observes that patients become more apt at assessing their own health status by seeking for information on the Internet, should be suspicious about whether this leads to less overprescribing, and makes patients worse off. In the situations identified above where small improvements in private patient information are detrimental, this does not mean that the patient will not seek additional information. Even if the individual patient knows that this will lead to a worse outcome, the individual patient cannot commit himself to not seeking such information, as for any *given* prescription strategy of the physician, the patient is better off with such information. Finally, a government which observes that physicians are making information available to patients that allows them to better assess their own status, should realize that this may involve *information pushing*, where the information made available to the patients makes them worse off. Since, as described, an improvement in the information of the patient may make the patient worse off and the physician better off, the physician then has an incentive to make information leading to such particular improvements available to the patient.

From the perspective of the game-theoretic literature on the value of private information, the paper has added to the literature one more example of a game where the value of private information may be negative. Consider in general a simultaneous-move trust game, where a trustor may either trust or not trust a trustee, and where the trustee may either be honest or dishonest (see [17]). When the trustor trusts the trustee, it is a best response for the trustee to be dishonest. When the trustee is dishonest, it is a best response for the trustor not to trust her. When the trustor does not trust, it is a best response for the trustee to be honest. Finally, when the trustee is honest, it is a best response for the trustor to trust. It follows that the only equilibrium in such a game is a mixed equilibrium, where the trustor randomizes between trusting and not trusting, and the trustee randomizes between being honest and not being honest. In line with our paper, one can construct an extension of the trust game, where there are two types of trustees, differing according to their trustworthiness, so that in equilibrium, the trustworthy trustee is honest, whereas the untrustworthy trustee is sometimes dishonest. Suppose further that the trustor gets an imperfect, private cue of the trustees' trustworthiness, and decides based on this cue whether or not to trust the trustee, so that in equilibrium the trustor with a cue of untrustworthiness of the trustee does not trust, whereas the trustor with a cue of trustworthiness of the trustee randomizes about whether or not to trust. Let the trustor's cue of the (un)trustworthiness of the trustee now become more accurate. Then in the new mixed equilibrium, the untrustworthy trustee will need to be dishonest more often to still keep the trustor with a cue of trustworthiness indifferent about whether to trust or not. Therefore, better information for the trustor about the trustee's (un)trustworthiness in this case makes the trustor worse off.

It should be stressed that such negative value of private information is not due to any sort of psychological distress of knowing what will happen. Following recent advances in behavioral economics and behavioral game theory, future research should consider the psychology of patients (for applications in health economics, see for example, [18, 19]. Further, it may be questioned whether patients and physicians are as sophisticated as is required for our results. New behavioral models can again be applied to take a lack of sophistication into account. Our model then provides a useful benchmark for assessing the effect of having players that are more behaviorally realistic.

## Figures and Tables

**Figure 7:**
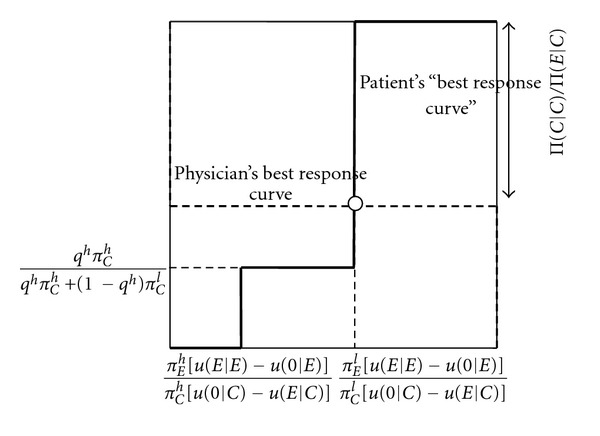
Low-type patient indifferent between accepting or refusing. *p* (probability that patient does not buy treatment when getting an unnecessary expensive treatment recommended). *q* (probability that the physician recommends an unnecessary expensive treatment).

**Figure 8:**
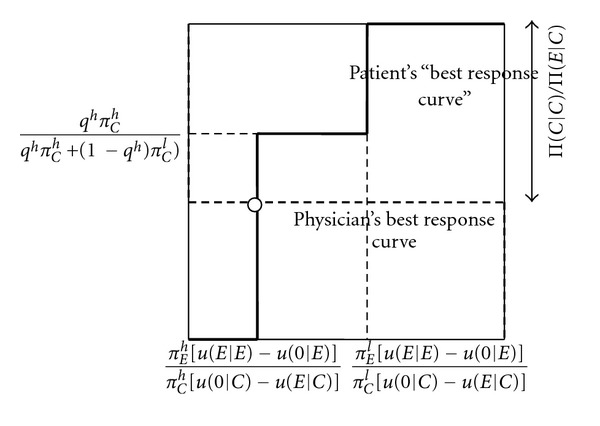
High-type patient indifferent between accepting or refusing. *p* (probability that patient does not buy treatment when getting an unnecessary expensive treatment recommended). *q* (probability that the physician recommends an unnecessary expensive treatment).

## Endnotes

1. A concrete example of the negative value of public information in the context of health economics is found in [20]. Consider a group of risk-averse individuals cooperating to set up a non-profit health insurance scheme. While self-interested, the individuals have an incentive to cooperate, as they do not know whether they will be ill or healthy. Assume now that through some sophisticated genetic testing, it becomes public information who will be ill and who will be healthy. Then the healthy individuals do not have any incentive to pay for the ill, and the health insurance scheme breaks down. Therefore, ex ante, before the results of the genetic tests become public information, the risk-averse individuals prefer that the results of the tests do not become public information, as they know it will destroy insurance. The literature further contains games where private information has negative value, but no concrete real-world applications are provided.

2. Following Xie et al. [7], one can also state this as there being two types of patients, in the terms of our model a patient who observes a *C* cue, and a patient who observes an *E* cue.

3. If it is instead assumed that the physician also observe the patient's cue, so that this cue is public information, a simple extension of de Jaegher and Jegers [4] is obtained, where the physician may be seen as playing a separate game with the patient with a *C* cue, and a separate game with the patient with an *E* cue, where she overprescribes more to the patient with the latter cue. In this case, an improvement in the quality of the patient's cues never makes the patient worse off.

4. This follows the set-up of the game in de Jaegher and Jegers [4]. In Xie et al. [7], the patient can only decide whether to accept or refuse the treatment proposed by the physician. The latter slightly simplifies the analysis, but does not modify our results. We keep de Jaegher and Jegers' setting here, because we can the show that the results do not depend on the assumption that the patient can only accept or refuse the prescribed treatment.

5. If Π(*C* | *C*)/Π(*E* | *C*) = Π(*C* | *E*)/Π(*E* | *E*), as considered by Xie et al. [7], in the mixed strategy equilibrum of the game, which is the only equilibrium where the patient does not always take the same action, the physician is not only indifferent about which treatment to prescribe in state *C*, but also in state *E*. If Π(*C* | *C*)/Π(*E* | *C*) < Π(*C* | *E*)/Π(*E* | *E*), it is easy to see that the physician either only prescribes the *C* treatment, only prescribes the *E* treatment, or systematically prescribes the wrong treatment in each state. It can be checked that under these circumstances, in every equilibrium, the patient always takes the same action in every state.

6. In terms of the taxonomy of information presented by Levine and Ponssard [10], while the patient's information is *private*, it is not *secret* information, in that the physician knows that the patient holds private information.

7. The mixed equilibrium is not the only perfect Bayesian equilibrium. As is the case for many games, the game has multiple equilibria. In particular, a set of equilibria exists where the patient never buys treatment, and where the physician's recommendations are sufficiently uninformative to justify this decision. If the patient believes that the physician's recommendations are never sufficiently informative, it is a best response for him not to buy treatment, whatever the physician recommends. Given this response by the patient, any response by the physician is a best response, including giving uninformative recommendations. Both players are better off, however, in the mixed equilibrium, and we assume here that players are able to coordinate on the mixed equilibrium.

8. Again, it can be shown that under the given assumptions, these are the only informative equilibria.

9. If the physician would be able to directly reveal information about the patient's state of the world, by making evidence available that is interpretable to the patient, the conflict of interest between the physician would not be an issue. Applying an argument by Milgrom [21], the patient should then interpret any lack of evidence as an indication that he does not need an expensive treatment, as a physician who does observe that the patient needs an expensive treatment may as well reveal this.

## Appendices

### A. Proofs

#### A.1. Proof of Proposition 1

To check for the existence of such a mixed equilibrium, note first that for any physician strategy where she never recommends the *C* treatment in state *E*, it is a best response for a patient who receives a *C* recommendation to always buy the *C* treatment, given that the physician never gives a *C* recommendation in state *E*. Further, denote by *q* the probability that the physician prescribes treatment *E* in state *C*. The patient who observes a cue *k* = *C*, *E* and who receives an *E* recommendation prefers buying the *E* treatment to buying no treatment if and only if:

(A.1) πCπ(k ∣ C)qπCπ(k ∣ C)q+πEπ(k ∣ E)u(E ∣ C) +πEπ(k ∣ E)πCπ(k ∣ C)q+πEπ(k ∣ E)u(E ∣ E)≥πCπ(k ∣ C)qπCπ(k ∣ C)q+πEπ(k ∣ E)u(0 ∣ C) +πEπ(k ∣ E)πCπ(k ∣ C)q+πEπ(k ∣ E)u(0 ∣ E)⇔q≤πEπ(k ∣ E)[u(E ∣ E)−u(0 ∣ E)]πCπ(k ∣ C)[u(0 ∣ C)−u(E ∣ C)],

where in the case without information, *π*(*k* | *E*) = *π*(*k* | *C*). Given the assumption that the patient without information prefers not to buy any treatment to buying the *E*-treatment (*π*_*E*_[*u*(*E* | *E*) − *u*(0 | *E*)]/*π*_*C*_[*u*(0 | *C*) − *u*(*E* | *C*)] < 1), for *q* smaller than this level, the patient prefers to do *E* to 0, and for *q* above this level, she prefers to do 0 to *E*.

The patient who observes a cue *i* = *C*, *E* and who receives an *E* recommendation prefers not buying any treatment to buying the *C* treatment if and only if:

(A.2) πCπ(k ∣ C)qπCπ(k ∣ C)q+πEπ(k ∣ E)u(C ∣ C) +πEπ(k ∣ E)πCπ(k ∣ C)q+πEπ(k ∣ E)u(C ∣ E)≤πCπ(k ∣ C)qπCπ(k ∣ C)q+πEπ(k ∣ E)u(0 ∣ C) +πEπ(k ∣ E)πCπ(k ∣ C)q+πEπ(k ∣ E)u(0 ∣ E)⇔q≤πEπ(k ∣ E)[u(0 ∣ E)−u(C ∣ E)]πCπ(k ∣ C)[u(C ∣ C)−u(0 ∣ C)],

where again in the case without patient information, *π*(*k* | *E*) = *π*(*k* | *C*).

Given the assumption that the patient without information prefers not to buy any treatment to buying the *C* treatment (*π*_*E*_[*u*(0 | *E*) − *u*(*C* | *E*)]/*π*_*C*_[*u*(*C* | *C*) − *u*(0 | *C*)] > 1), (A.2) is always valid for *π*(*i* | *E*) = *π*(*i* | *C*). Rounding up, for *q* < *π*_*E*_[*u*(*E* | *E*) − *u*(0 | *E*)]/*π*_*C*_[*u*(0 | *C*) − *u*(*E* | *C*)], the patient prefers *E* to 0, and 0 to *C*. For *q* = *π*_*E*_[*u*(*E* | *E*) − *u*(0 | *E*)]/*π*_*C*_[*u*(0 | *C*) − *u*(*E* | *C*)], the patient is indifferent between *E* and 0 (and therefore weakly prefers to do 0 with probability *p* when getting an *E* recommendation), and prefers 0 to *C*. For *q* > *π*_*E*_[*u*(*E* | *E*) − *u*(0 | *E*)]/*π*_*C*_[*u*(0 | *C*) − *u*(*E* | *C*)], the patient prefers 0 to *E* and 0 to *C*. These best responses of the patient as a function of *q* are summarized in the best response curve of the patient depicted Figure 1 (solid lines).

Denote by *p*_*C*_ be the probability that, from the perspective of the physician, the patient does 0 upon receiving an *E* recommendation in state *C*. Given this *p*_*C*_, the physician prefers to give a *C* recommendation in state *C* if and only if:

(A.3) $$\begin{array}{c} \Pi \left(C\;|\;C\right)\geq \left(1-p_{C}\right)\Pi \left(E\;|\;C\right)\Leftrightarrow \left(1-p_{C}\right)\leq \frac{\Pi \left(C\;|\;C\right)}{\Pi \left(E\;|\;C\right)}. \end{array}$$

Denote by *p*_*E*_ the probability that, from the perspective of the physician, the patient does 0 upon receiving an *E* recommendation in state *E*. The physician prefers to prescribe the *C* treatment in state *E* if:

(A.4) $$\begin{array}{c} \Pi \left(C\;|\;E\right)\geq \left(1-p_{E}\right)\Pi \left(E\;|\;E\right)\Leftrightarrow \left(1-p_{E}\right)\leq \frac{\Pi \left(C\;|\;E\right)}{\Pi \left(E\;|\;E\right)}. \end{array}$$

If the patient does not receive different information in states *C* and *E*, *p*_*C*_ and *p*_*E*_ need to be equal, so that we can denote*p* = *p*_*C*_ = *p*_*E*_. Given our assumption that 1 > Π(*C* | *C*)/Π(*E* | *C*) > Π(*C* | *E*)/Π(*E* | *E*), for (1 − *p*) > Π(*C* | *C*)/Π(*E* | *C*), the physician prefers to recommend *E* in both states. For (1 − *p*) = Π(*C* | *C*)/Π(*E* | *C*), she is indifferent between prescribing *E* and *C* in state *C* (and therefore weakly prefers to prescribe *E* with probability *q*) and prefers to recommend *E* in state *E*. For Π(*C* | *C*)/Π(*E* | *C*) > (1 − *p*) > Π(*C* | *E*)/Π(*E* | *E*), she recommends the efficient treatment. For (1 − *p*) = Π(*C* | *E*)/Π(*E* | *E*), she recommends *C* in state *C*, and is indifferent about whether or not to recommend *C* in state *E*. Finally, for (1 − *p*) < Π(*C* | *E*)/Π(*E* | *E*), she always recommends the *C* treatment. These best responses of the physician as a function of *p* are depicted in Figure 1 by means of the physician's best response curve (dashed lines), which gives the probability that the physician recommends *E* in state *C*. As illustrated in Figure 1, the mixed equilibrium is found by the intersection point of the two best response curves, where (1 − *p*) = Π(*C* | *E*)/Π(*E* | *E*) and *q* = *π*_*E*_[*u*(*E* | *E*) − *u*(0 | *E*)]/*π*_*C*_[*u*(0 | *C*) − *u*(*E* | *C*)].

#### A.2. Proof of Proposition 2

We again note that for the specified mixed strategy of the physician, the patient who receives a *C* recommendation always buys *C*. We continue to consider the best response of the patient upon an *E* recommendation. Given the assumption that *π*_*i*_ *π*(*k* | *i*)[*u*(*i* | *i*) − *u*(0 | *i*)]/*π*_*j*_ *π*(*k* | *j*)[*u*(0 | *j*) − *u*(*i* | *j*)] < 1, by (A.1) and (A.2), by the same reasoning as in the proof of Proposition 1, for each cue *k* observed by the patient, there is a critical level of *q* such that for all *q* strictly below this critical level, the patient with a cue *k* prefers to do *E*; for *q* equal to the critical level, the patient is indifferent between doing *E* or 0, and strictly prefers not to do *C*; for *q* above the critical level, she strictly prefers to do 0. Further, given that *π*(*C* | *E*)/*π*(*C* | *C*) < *π*(*E* | *E*)/*π*(*E* | *C*), the critical level of *q* (as given by the right-hand side of (A.1)) for the *C* cue lies below the critical level for the *E* cue.

As summarized by the patient's best response curves in Figures 2 and 3, it follows that for *q* < *π*_*E*_ *π*(*C* | *E*)[*u*(*E* | *E*) − *u*(0 | *E*)]/*π*_*C*_ *π*(*C* | *C*)[*u*(0 | *C*) − *u*(*E* | *C*)], the patient receiving an *E* recommendation strictly prefers not to buy treatment both when having received a *C* cue or an *E* cue. For *q* = *π*_*E*_ *π*(*C* | *E*)[*u*(*E* | *E*) − *u*(0 | *E*)]/*π*_*C*_ *π*(*C* | *C*)[*u*(0 | *C*) − *u*(*E* | *C*)], the patient receiving an *E* recommendation is indifferent between doing *E* and doing 0 when having received a *C* cue (where *E* and 0 are both strictly preferred to buying the *C* treatment), and strictly prefers to do *E* when having received an *E* cue. It follows that the probability that the patient refuses an *E* recommendation in state *C* is anywhere between 0 and *π*(*C* | *C*). For *π*_*E*_ *π*(*C* | *E*)[*u*(*E* | *E*) − *u*(0 | *E*)]/*π*_*C*_ *π*(*C* | *C*)[*u*(0 | *C*) − *u*(*E* | *C*)] < *q*<*π*_*E*_ *π*(*E* | *E*)[*u*(*E* | *E*) − *u*(0 | *E*)]/*π*_*C*_ *π*(*E* | *C*)[*u*(0 | *C*) − *u*(*E* | *C*)], the patient receiving an *E* recommendation strictly prefers to do 0 when having received a *C* cue, and strictly prefers to do *E* when having received an *E* cue. It follows that the probability that the patient refuses an *E* recommendation in state *C* is exactly *π*(*C* | *C*). For *q* = *π*_*E*_ *π*(*E* | *E*)[*u*(*E* | *E*) − *u*(0 | *E*)]/*π*_*C*_ *π*(*E*|*C*)[*u*(0 | *C*) − *u*(*E* | *C*)], the patient receiving an *E* recommendation strictly prefers to do 0 when having received a *C* cue, and is indifferent between doing 0 or *E* when having received an *E* cue (where both of these actions are preferred to *C*). It follows that the probability that the patient refuses an *E* recommendation in state *C* is anywhere between *π*(*C* | *C*) and 1. Finally, when *q* > *π*_*E*_ *π*(*E* | *E*)[*u*(*E* | *E*) − *u*(0 | *E*)]/*π*_*C*_ *π*(*E* | *C*)[*u*(0 | *C*) − *u*(*E* | *C*)], the patient getting an *E* recommendation strictly prefers 0 upon any cue. These results are illustrated by the patient's best response curve depicted in Figures 2 and 3, where this curve is here represented as the probability *p*_*C*_, as a function of the probability *q* that the physician prescribes *E* in state *C*, that a patient does 0 when receiving an *E* recommendation in state *C*.

Consider now a patient who, from the perspective of the physician, when receiving an *E* recommendation in state *C*, does 0 with probability *p*_*C*_ and *E* with probability (1 − *p*_*C*_). Then the physician strictly prefers to recommend *C* in state *C* if (A.3) is valid. Denote by *r*_*C*_ the probability that the patient does 0 when obtaining an *E* recommendation after having observed a *C* cue, and by *r*_*E*_ the probability that the patient does 0 when obtaining an *E* recommendation after having observed an *E* cue. Then, as shown above, there are several ways in which the patient can mix between doing 0 or *E* when getting an *E* recommendation. Each time, we look under which condition such a way of mixing by the patient makes the physician indifferent between recommending *C* and *E* in state *C*.

First, the patient receiving an *E* recommendation may mix when having observed a *C* cue, and may always do *E* when observing an *E* cue. In this case, in a mixed equilibrium we have that (1 − *p*_*C*_) = *π*(*C* | *C*)(1 − *r*_*C*_) + *π*(*E* | *C*). Given that it must be the case that 0 < (1 − *r*_*C*_) < 1, and by the fact that in the mixed equilibrium by (A.3) it must be that (1 − *p*_*C*_) = Π(*C* | *C*)/Π(*E* | *C*)  , this case is only possible if (1 − *r*_*C*_) = Π(*C* | *C*)Π(*E* | *C*)^−1^ *π*(*C* | *C*)^−1^−*π*(*E* | *C*)*π*(*C* | *C*)^−1^ > 0 meaning that it must be the case that Π(*C* | *C*)Π(*E* | *C*)^−1^ > *π*(*E* | *C*). Note further that (1 − *r*_*C*_) < 1 as Π(*C* | *C*)Π(*E* | *C*)^−1^ < 1. Also, note that (1 − *p*_*E*_) = *π*(*C* | *E*)(1 − *r*_*C*_) + *π*(*E* | *E*)>(1 − *p*_*C*_) = *π*(*C* | *C*)(1 − *r*_*C*_) + *π*(*E* | *C*) given that *π*(*C* | *C*) > *π*(*E* | *C*). Since Π(*C* | *C*)/Π(*E* | *C*) > Π(*C* | *E*)/Π(*E* | *E*) and (1 − *p*_*C*_) = Π(*C* | *C*)/Π((*E* | *C*), it follows that (1 − *p*_*E*_)>(1 − *p*_*C*_) = Π(*C* | *C*)/Π(*E* | *C*) > Π(*C*|*E*)/Π(*E* | *E*), so that by (A.4) the physician in state *E* strictly prefers to prescribe the *E* treatment. This case is depicted in Figure 2, where one finds the best response curve of the physician as a function of *p*_*C*_. Combining with the best response curve of the patient, it can be seen that the patient must now randomize when observing a *C* cue.

Second, the patient receiving an *E* recommendation may always do 0 when observing a *C* cue, and may always do *E* when observing an *E* cue. In this case, in a mixed equilibrium (1 − *p*_*C*_) = *π*(*E* | *C*). Given (A.3), the physician is now only indifferent between recommending *E* and 0 in state *C* if it happens to be exactly the case that Π(*C* | *C*)/Π(*E* | *C*) = *π*(*E* | *C*). Further, in this case (1 − *p*_*E*_) = *π*(*E* | *E*)>(1 − *p*_*C*_) = *π*(*E* | *C*). Again, given that Π(*C* | *C*)/Π(*E* | *C*) > Π(*C* | *E*)/Π(*E* | *E*) and (1 − *p*_*C*_) = Π(*C* | *C*)/Π(*E* | *C*), it follows that (1 − *p*_*E*_)>(1 − *p*_*C*_) = Π(*C* | *C*)/Π(*E* | *C*) > Π(*C* | *E*)/Π(*E* | *E*), so that by (A.4) the physician in state *E* strictly prefers to prescribe the *E* treatment. This border case is not considered in the proposition.

Third, the patient receiving an *E* recommendation may always do 0 when observing a *C* cue, and may mix between doing 0 and *E* when observing an *E* cue. In this case, in a mixed equilibrium we have that (1 − *p*_*C*_) = (1 − *r*_*E*_)*π*(*E* | *C*). Given that it must be the case that 0 < (1 − *r*_*C*_) < 1, and by the fact that in the mixed equilibrium by (A.3) it must be that (1 − *p*_*C*_) = Π(*C* | *C*)/Π(*E* | *C*), this case is only possible if (1 − *r*_*E*_) = Π(*C* | *C*)Π(*E* | *C*)^−1^ *π*(*E* | *C*)^−1^ meaning that it must be the case that Π(*C* | *C*)Π(*E* | *C*)^−1^ < *π*(*E* | *C*). Also, note that (1 − *p*_*E*_) = (1 − *r*_*E*_)*π*(*E* | *E*)>(1 − *p*_*C*_) = (1 − *r*_*E*_)*π*(*E* | *C*) given that *π*(*E* | *E*) > *π*(*E* | *C*). Since Π(*C* | *C*)/Π(*E* | *C*) > Π(*C* | *E*)/Π(*E* | *E*) and (1 − *p*_*C*_) = Π(*C* | *C*)/Π(*E* | *C*), it follows that (1 − *p*_*E*_) > (1 − *p*_*C*_) = Π(*C* | *C*)/Π(*E* | *C*) > Π(*C* | *E*)/Π(*E* | *E*), so that by (A.4) the physician in state *E* strictly prefers to prescribe the *E* treatment. This case is depicted in Figure 3, where one finds the best response curve of the physician as a function of *p*_*C*_. Combining with the best response curve of the patient, it can be seen that the patient must now randomize when observing an *E* cue.

#### A.3. Proof to Proposition 3

(i) Consider first the mixed equilibrium of type (i) in Proposition 2. As a function of *q* and *r*_*C*_, the patient's expected payoff in such an equilibrium equals

(A.5) πC{q[π(C ∣ C)(rCu(0 ∣ C)+(1−rC)u(E ∣ C))  +π(E ∣ C)u(E ∣ C)]+(1−q)u(C ∣ C)} +πE{π(C ∣ E)(rCu(0 ∣ E)+(1−rC)u(E ∣ E))    +π(E ∣ E)u(E ∣ E)}

which can be rewritten as

(A.6) rC{πCπ(C ∣ C)qu(E ∣ C)+πEπ(C ∣ E)u(E ∣ E)} +(1−rC){πCπ(C ∣ C)qu(0 ∣ C)+πEπ(C ∣ E)u(0 ∣ E)} +πCqπ(E ∣ C)u(E ∣ C)+πC(1−q)u(C ∣ C) +πEπ(E ∣ E)u(E ∣ E).

Using (A.1) for the case where the patient observes a *C* cue, and canceling out the denominators, we have

(A.7) πCπ(C ∣ C)qu(0 ∣ C)+πEπ(C ∣ E)u(0 ∣ E)  =πCπ(C ∣ C)qu(E ∣ C)+πEπ(C ∣ E)u(E ∣ E).

Plugging the latter into the rewritten expected payoff of the patient, we obtain that the patient's expected payoff equals

(A.8) rC{πCπ(C ∣ C)  qu(E ∣ C)+πEπ(C ∣ E)u(E ∣ E)} +(1−rC){πCπ(C ∣ C)qu(E ∣ C)+πEπ(C ∣ E)u(E ∣ E)} +πCqπ(E ∣ C)u(E ∣ C)+πC(1−q)u(C ∣ C) +πEπ(E ∣ E)u(E ∣ E)

which in turn equals

(A.9) $$\begin{array}{c} \pi_{C}\left\{qu\left(E\;|\;C\right)+\left(1-q\right)u\left(C\;|\;C\right)\right\}+\pi_{E}u\left(E\;|\;E\right) \end{array}$$

with

(A.10) $$\begin{array}{c} q=\frac{\pi_{E}\pi \left(C\;|\;E\right)\left[u\left(E\;|\;E\right)-u\left(0\;|\;E\right)\right]}{\pi_{C}\pi \left(C\;|\;C\right)\left[u\left(0\;|\;C\right)-u\left(E\;|\;C\right)\right]}. \end{array}$$

An increase in patient information decreases *π*(*C* | *E*) and increases *π*(*C* | *C*), and therefore leads to a decrease in *q*. Note further that as *π*(*E* | *C*) decreases, it continues to be the case that Π(*C* | *C*)/Π(*E* | *C*) > *π*(*E* | *C*), so that the type of the mixed equilibrium cannot change. By (A.9), it follows that the patient's payoff increases. An increase in the patient's information such that he is perfectly informed of the state of the world leads to a new equilibrium where the patient buys the efficient treatment in each state, and yields the patient a payoff of *π*_*C*_ *u*(*C* | *C*) + *π*_*E*_ *u*(*E* | *E*), which is also larger than what we have in (A.9).

As a function of *q* and *r*_*C*_, the physician's expected payoff in the equilibrium under Proposition 2(i) equals

(A.11) πC{q[π(C ∣ C)(1−rC)+π(E ∣ C)]Π(E ∣ C)  +(1−q)Π(C ∣ C)} +πE{π(C ∣ E)(1−rC)Π(E ∣ E)+π(E ∣ E)Π(E ∣ E)}.

We know by (A.3) and from the proof of Proposition 2 that in the specified mixed equilibrium, (1 − *p*_*C*_)Π(*E* | *C*) = Π(*C* | *C*), where (1 − *p*_*C*_) = *π*(*C* | *C*)(1 − *r*_*C*_) + *π*(*E* | *C*). Substituting into the physician's profits, we obtain that these equal

(A.12) πCΠ(C ∣ C) +πE{π(C ∣ E)(1−rC)Π(E ∣ E)+π(E ∣ E)Π(E ∣ E)},

where (1 − *r*_*C*_) = Π(*C* | *C*)Π(*E* | *C*)^−1^ *π*(*C* | *C*)^−1^ − *π*(*C* | *C*)^−1^ + 1.

Note now that

(A.13) ∂(1−rC)∂π(C ∣ C)  =−Π(C ∣ C)Π(E ∣ C)−1π(C ∣ C)−2+π(C ∣ C)−2>0.

An increase in the patient's information increases *π*(*E* | *E*) and *π*(*C* | *C*) and decreases *π*(*C* | *E*). As (1 − *r*_*C*_)Π(*E* | *E*) < Π(*E* | *E*), holding (1 − *r*_*C*_) fixed, an increase in information leads to more weight to the larger term Π(*EE*) and less weight to the smaller term (1 − *r*_*C*_)Π(*E* | *E*). The increase in (1 − *r*_*C*_) further means that the smaller term itself becomes larger. It follows that the physician's payoff increases. The same applies when the patient obtains perfect information, so that the physician's expected payoff becomes *π*_*C*_Π(*C* | *C*) + *π*_*E*_Π(*E* | *E*), which is larger than the expression in (A.12).

(ii) Consider next the equilibrium of type (ii) in Proposition 2. As a function of *q* and *p*_*C*_, the patient's expected payoff in such an equilibrium equals

(A.14) πC{q[π(C ∣ C)u(0 ∣ C)+π(E ∣ C)  ×(rEu(0 ∣ C)+(1−rE)u(E ∣ C))]+(1−q)u(C ∣ C)}  +πE{π(C ∣ E)u(0 ∣ E)     +π(E ∣ E)(rEu(0 ∣ E)+(1−rE)u(E ∣ E))}.

We can rewrite this as:

(A.15) rE{πCπ(E ∣ C)qu(0 ∣ C)+πEπ(E ∣ E)u(0 ∣ E)} +(1−rE){πCπ(E ∣ C)qu(E ∣ C)+πEπ(E ∣ E)u(E ∣ E)} +πCqπ(C ∣ C)u(0 ∣ C)+πC(1−q)u(C ∣ C) +πEπ(C ∣ E)u(0 ∣ E).

Using (A.1) for the case where the patient observes a *C* cue, and canceling out the denominators, we have

(A.16) πCπ(E ∣ C)qu(E ∣ C)+πEπ(E ∣ E)u(E ∣ E)  =πCπ(E ∣ C)qu(0 ∣ C)+πEπ(E ∣ E)u(0 ∣ E).

Plugging the latter into the rewritten expected payoff of the patient, we obtain that the patient's expected payoff equals

(A.17) rE{πCπ(E ∣ C)qu(0 ∣ C)+πEπ(E ∣ E)u(0 ∣ E)} +(1−rE){πCπ(E ∣ C)qu(0 ∣ C)+πEπ(E ∣ E)u(0 ∣ E)} +πCqπ(C ∣ C)u(0 ∣ C)+πC(1−q)u(C ∣ C) +πEπ(C ∣ E)u(0 ∣ E)

which in turn equals

(A.18) $$\begin{array}{c} \pi_{C}\left\{qu\left(0\;|\;C\right)+\left(1-q\right)u\left(C\;|\;C\right)\right\}+\pi_{E}u\left(0\;|\;E\right), \end{array}$$

where

(A.19) $$\begin{array}{c} q=\frac{\pi_{E}\pi \left(E\;|\;E\right)\left[u\left(E\;|\;E\right)-u\left(0\;|\;E\right)\right]}{\pi_{C}\pi \left(E\;|\;C\right)\left[u\left(0\;|\;C\right)-u\left(E\;|\;C\right)\right]}. \end{array}$$

An increase in patient information decreases *π*(*E* | *C*) and increases *π*(*E* | *E*), and therefore leads to an increase in *q*. Note further that as *π*(*E* | *C*) decreases, it need no longer be the case that Π(*C* | *C*)/Π(*E* | *C*) < *π*(*E* | *C*), so that the type of the mixed equilibrium may change. We first consider small increases in information, such that the same type of mixed equilibrium is maintained. By (A.18), it follows that the patient's payoff *decreases* with a small increase in his information. The same does not apply with a large increase in the patient's information. Consider the extreme case where the patient obtains perfect information. Then in the newly informed equilibrium the physician is forced to recommend the efficient treatment, and the patient obtains expected payoff *π*_*C*_ *u*(*C* | *C*) + *π*_*E*_ *u*(*E* | *E*), which is larger than (A.18).

As a function of *q* and *p*_*C*_, the physician's expected payoff in the equilibrium under Proposition 2(ii) equals

(A.20) πC{qπ(E ∣ C)(1−rE)Π(E ∣ C)+(1−q)Π(C ∣ C)}  +πEπ(E ∣ E)(1−rE)Π(E ∣ E).

We know by (A.3) and from the proof of Proposition 2 that in the specified mixed equilibrium, (1 − *p*_*C*_)Π(*E* | *C*) = Π(*C* | *C*), where (1 − *p*_*C*_) = (1 − *r*_*E*_)*π*(*E* | *C*). Substituting into the physician's profits, we obtain that these equal

(A.21) $$\begin{array}{c} \pi_{C}\Pi \left(C\;|\;C\right)+\pi_{E}\pi \left(E\;|\;E\right)\left(1-r_{E}\right)\Pi \left(E\;|\;E\right), \end{array}$$

where (1 − *r*_*E*_) = Π(*C* | *C*)Π(*E* | *C*)^−1^ *π*(*E* | *C*)^−1^.

A small increase in patient information decreases *π*(*E* | *C*) and increases *π*(*E* | *E*), and therefore leads to a decrease in *q*. It follows by (A.21) that the physician's payoff increases with a small increase in information of the patient. An increase in the patient's information such that he is perfectly informed of the state of the world yields the physician a payoff of *π*_*C*_Π(*C* | *C*) + *π*_*E*_Π(*E* | *E*), which is also larger than what we have in (A.21).

For a bigger change in the patient's information, the type of mixed equilibrium changes from an equilibrium of type (ii) to an equilibrium of type (i). Denote the mixing probabilities and probabilities of the cues with a “*” after information has improved. For the patient, the improved information with a change in the type of the mixed equilibrium means obtaining *π*_*C*_{*q***u*(*E* | *C*)+(1 − *q**)*u*(*C* | *C*)} + *π*_*E*_ *u*(*E* | *E*), with *q** = *π*_*E*_ *π*(*C* | *E*)*[*u*(*E* | *E*) − *u*(0 | *E*)]/*π*_*C*_ *π*(*C* | *C*)*[*u*(0 | *C*) − *u*(*E* | *C*)], rather than *π*_*C*_{*qu*(0 | *C*)+(1 − *q*)*u*(*C* | *C*)} + *π*_*E*_ *u*(0 | *E*), with *q* = *π*_*E*_ *π*(*E* | *E*)[*u*(*E* | *E*) − *u*(0 | *E*)]/*π*_*C*_ *π*(*E* | *C*)[*u*(0 | *C*) − *u*(*E* | *C*)], where *q** < *q*.

The patient is better off now with more information as:

(A.22) πC{qu(0 ∣ C)+(1−q)u(C ∣ C)}+πEu(0 ∣ E)<πC{q∗u(E ∣ C)+(1−q∗)u(C ∣ C)}+πEu(E ∣ E)⇔πCq∗[u(C ∣ C)−u(E ∣ C)] −πCq[u(C ∣ C)u(0 ∣ C)]<πE[u(E ∣ E)−u(0 ∣ E)]⇔πCq∗[u(0 ∣ C)−u(E ∣ C)]−πC(q−q∗) ×[u(C ∣ C)−u(0 ∣ C)]<πE[u(E ∣ E)−u(0 ∣ E)]⇔−πC(q−q∗)[u(C ∣ C)−u(0 ∣ C)]<πE[1−π(C ∣ E)∗π(C ∣ C)∗][u(E ∣ E)−u(0 ∣ E)].

After improved patient information, the physician obtains *π*_*C*_Π(*C* | *C*)+*π*_*E*_{*π*(*C* | *E*)*(1 − *r*_*C*_*)Π(*E* | *E*) + *π*(*E* | *E*)*Π(*E* | *E*)} instead of *π*_*C*_Π(*C* | *C*) + *π*_*E*_ *π*(*E* | *E*)(1 − *r*_*E*_)Π(*E* | *E*), where clearly the situation for the physician after improvement of the patient's information is better.

### B. Summary of the Results of Xie et al. [7]

Xie et al. [7] present an extension of the model of de Jaegher and Jegers [4] reviewed in Section 3.1. Instead of one type of patient, there are now two possible type of patients, distinguished by the probability that they are in state *C*. With probability *q*_*h*_ the patient is of type *h*, and then has a high probability of being in state *C*, *π*_*C*_^*h*^, and a low probability of being in state *E*, *π*_*E*_^*h*^, with *π*_*C*_^*h*^ + *π*_*E*_^*h*^ = 1. With probability (1 − *q*_*h*_) the patient is of type *l*, and has a low probability of being in state *C*, *π*_*C*_^*l*^, and a high probability of being in state *E*, *π*_*E*_^*l*^, with *π*_*C*_^*l*^ + *π*_*E*_^*l*^ = 1, where *π*_*C*_^*h*^ > *π*_*C*_^*l*^. The physician knows the state, but does not know the patient's type, whereas the patient knows his own type, but not the state. From the perspective of a physician who observes state *C*, the probability that the patient follows the *E* recommendation, as a function of the probability *q* that the physician prescribes the *E* treatment in state *C*, is depicted in Figure 7. For *q* < *π*_*E*_^*h*^[*u*(*E* | *E*) − *u*(0 | *E*)]/*π*_*C*_^*h*^[*u*(0 | *C*) − *u*(*E* | *C*)], both types of patients follow the *E* recommendation. At *q* = *π*_*E*_^*h*^[*u*(*E* | *E*) − *u*(0 | *E*)]/*π*_*C*_^*h*^[*u*(0 | *C*) − *u*(*E* | *C*)], the type *h* patient is just indifferent about whether or not to follow an *E* recommendation, and by the fact that *π*_*C*_^*h*^ > *π*_*C*_^*l*^, the type *l* patient prefers to follow. This means that from the perspective of the physician observing state *C*, the probability that the patient does not follow lies anywhere between 0 and *q* ^*h*^ *π*_*C*_^*h*^/(*q* ^*h*^ *π*_*C*_^*h*^ + (1 − *q* ^*l*^)*π*_*C*_^*l*^), the probability that the patient is of type *h* for a physician who has observed state *C*. For *q* such that *π*_*E*_^*h*^[*u*(*E* | *E*) − *u*(0 | *E*)]/*π*_*C*_^*h*^[*u*(0 | *C*) − *u*(*E* | *C*)] < *q* < *π*_*E*_^*l*^[*u*(*E* | *E*) − *u*(0 | *E*)]/*π*_*C*_^*l*^[*u*(0 | *C*) − *u*(*E* | *C*)], the type *h* patient does not follow the *E* recommendation and the type *l* patient follows the *E* recommendation, so that the physician who observes state *C* may expect that the patient does not follow with probability *q* ^*h*^ *π*_*C*_^*h*^/(*q* ^*h*^ *π*_*C*_^*h*^ + (1 − *q* ^*l*^)*π*_*C*_^*l*^). For *q* = *π*_*E*_^*l*^[*u*(*E* | *E*) − *u*(0 | *E*)]/*π*_*C*_^*l*^[*u*(0 | *C*) − *u*(*E* | *C*)], the type *l* patient is indifferent between following and not following an *E* recommendation, so that the probability of not following from the perspective of a physician who has observed state *C* lies between *q* ^*h*^ *π*_*C*_^*h*^/(*q* ^*h*^ *π*_*C*_^*h*^ + (1 − *q* ^*l*^)*π*_*C*_^*l*^) and 1. Finally, as *q* is still further increased, the patient does not follow an *E* recommendation with probability 1. These arguments yield what can still be termed the patient's “best response curve” in Figures 7 and 8, where the patient is then interpreted as an average patient. The physician's best response does not alter.

Figures 7 and 8 show the two cases obtained by Xie et al. If *q* ^*h*^ *π*_*C*_^*h*^/(*q* ^*h*^ *π*_*C*_^*h*^ + (1 − *q* ^*h*^)*π*_*C*_^*l*^) < 1 − Π(*C* | *C*)/Π(*E* | *C*), meaning that *q* ^*h*^ < (Π(*E* | *C*) − Π(*C* | *C*))/(Π(*C* | *C*)(*π*_*C*_^*h*^/*π*_*C*_^*l*^) + Π(*E* | *C*) − Π(*C* | *C*))(where the right-hand side is the expression denoted as *q** in Proposition 3 of Xie et al., [7, page 819], we obtain the case in Figure 7, where it is the type *h* patient who is indifferent between following and not following an expensive recommendation. In the complementary case, we obtain the case in Figure 8, where it is the type *l* patient who is indifferent between accepting and refusing.

Xie et al. now look at the effect of an increase in *π*_*C*_^*h*^, and of an increase in *π*_*C*_^*l*^, on the probability that the physician prescribes an unnecessary expensive treatment. An increase in *π*_*C*_^*h*^ does not affect the probability that the physician overprescribes in the case of Figure 7, but decreases the probability that the physician overprescribes in the case of Figure 8 (Xie et al., [7, Theorem 3, page 821]). Further, an increase in *π*_*C*_^*h*^ can be calculated to increase *q* ^*h*^ *π*_*C*_^*h*^/(*q* ^*h*^ *π*_*C*_^*h*^ + (1 − *q* ^*h*^)*π*_*C*_^*l*^) [7, Theorem 2, p.821]. As is clear then, if in the case in Figure 7 the increase in *π*_*C*_^*h*^ is sufficiently large, instead of *q* ^*h*^ *π*_*C*_^*h*^/(*q* ^*h*^ *π*_*C*_^*h*^ + (1 − *q* ^*h*^)*π*_*C*_^*l*^) continuing to be smaller than 1 − Π(*C* | *C*)/Π(*E* | *C*), it will become larger than 1 − Π(*C* | *C*)/Π(*E* | *C*), so that there is a switch (termed a “regime change” by the authors) from an equilibrium where the type *l* patient is indifferent, to the case where the type *h* is indifferent, leading to a smaller probability of overprescription. Thus, making it more likely that type *h* patients are in state *C* never leads to a reduction in the probability of overprescribing.

In the same manner, an increase in *π*_*C*_^*l*^ decreases the probability that the physician overprescribes in the case of Figure 7, but does not change the probability that the physician overprescribes in the case of Figure 8 (Xie et al., [7, Theorem 4, page 821]). Further, an increase in *π*_*C*_^*l*^ clearly decreases *q* ^*h*^ *π*_*C*_^*h*^/(*q* ^*h*^ *π*_*C*_^*h*^ + (1 − *q* ^*h*^)*π*_*C*_^*l*^) [7, Theorem 2, page 821]. As is clear from Figure 8, for a sufficiently large increase in *π*_*C*_^*l*^, instead of *q* ^*h*^ *π*_*C*_^*h*^/(*q* ^*h*^ *π*_*C*_^*h*^ + (1 − *q* ^*h*^)*π*_*C*_^*l*^) continuing to be larger than 1 − Π(*C* | *C*)/Π(*E* | *C*), it will become smaller than 1 − Π(*C* | *C*)/Π(*E* | *C*), leading to a regime switch from an equilibrium where the consumer of type *h* is indifferent, to an equilibrium where the consumer of type *l* is indifferent. This leads to an *increase* in the probability that the physician overprescribes [7, Theorem 4].

Xie et al. interpret the type *h* patient as a relatively well-informed patient, and the type *l* patient as a relatively ill-informed patient. An increase in *π*_*C*_^*h*^ (resp., *π*_*C*_^*l*^) is interpreted as an increase in the quality of the information of the relatively informed (respectively uninformed) patient. In this interpretation, the authors' results suggest that small increases in the information of patients can at most lead to a decrease in the probability that the physician overprescribes. The only manner in which, in Xie et al.'s interpretation of patient information, improved patient information increases the probability that the physician overprescribes, is if (A.1) it is currently the relatively well-informed patient who is indifferent between following or not following an expensive recommendation; (A.2) there is a substantial improvement of the information of the relatively uninformed patient, so that it is this patient who in equilibrium is indifferent between accepting or refusing treatment.

Particular about Xie et al.'s model is that a patient who is more likely to be in state *C* (i.e., a type *l* patient), and therefore more likely to need a cheap treatment, is interpreted as being better informed. Therefore, a change in the patient's information is at the same time a change in the incidence of disease. One can therefore also interpret Xie et al.'s results as saying that if the patients who are relatively more likely to need an expensive treatment, become less likely to need the expensive treatment, then this may actually lead to an increase in the probability that the physician overprescribes. In our model, on the contrary, the incidence of disease is given, but patients differ according to the quality of the noisy signals that they get about the incidence of disease.
